# Protective Effect of Ultraviolet C Irradiation on the Gastric Mucosa of Rats with Chronic Gastritis Induced by Physicochemical Stimulations

**DOI:** 10.1155/2021/5189797

**Published:** 2021-03-17

**Authors:** Likang Wang, Wei Chen, Xisheng Lin, Zhao Zhang, Na Wang, Youkui Lv, Xinglin Wang, Yueming Gao

**Affiliations:** ^1^Medical School of Chinese PLA, Beijing 100853, China; ^2^Department of Rehabilitation Medicine, The Third Medical Centre, Chinese PLA General Hospital, Beijing 100039, China; ^3^Department of Rehabilitation Medicine, The Second Medical Centre, Chinese PLA General Hospital, Beijing 100853, China

## Abstract

**Background:**

Chronic gastritis (CG) is a common digestive disease with the highest morbidity among multiple digestive diseases, which seriously lowers the life quality of patients. The pathological alternations of gastric mucosa, and its possible mechanisms have been the focus of CG-related researches. Accumulative basic and clinical evidence has confirmed that ultraviolet C (UVC) is effective in relieving superficial acute infective inflammation, skin and mucous membrane injuries, and ulcers, and promoting wound healing.

**Objective:**

This study was aimed at investigating the protective effects of UVC on gastric mucosal injury in rats stimulated with physicochemical irritants like ethanol and exploring the mechanisms underlying the protection by UVC against gastric mucosal injury and CG.

**Methods:**

Fifty Wistar rats were randomly divided into five groups, including Group A (normal), Group B (model), Group C (omeprazole treatment), Group D (intragastric UVC irradiation for 24 s × 2 yields), and Group E (intragastric UVC irradiation for 48 s × 2 yields). Rats in Groups B–E were made CG model by physicochemical stimulations. All rats were sacrificed one week after the 22-week experiment, and gastric tissues were harvested. Histopathological examinations were performed. The activities of superoxide dismutase and catalase as well as the contents of reduced glutathione and malondialdehyde in gastric mucosal tissues were detected. Serum interleukin-6, interleukin-1beta, tumor necrosis factor-alpha, pepsin, and gastrin were measured.

**Results:**

Results showed that physiochemical irritants like ethanol could be used for easily establishing a rat CG model that shared similar pathological features with human CG. Intragastric UVC irradiation could promote the repair of gastric mucosa and improve the atrophy of gastric mucosa by inhibiting the inflammatory factors, increasing the levels of pepsin and gastrin, decreasing the expression of lipid peroxide, and enhancing the activity of superoxide dismutase and catalase and the levels of reduced glutathione. UVC irradiation for 48 s × 2 yields showed the strongest protective effect.

**Conclusion:**

UVC irradiation could inhibit the inflammatory factors, activate the antioxidative system, and enhance the secretion of pepsin and gastrin, which promoted the repair of injured gastric mucosa and improved gastric mucosa atrophy.

## 1. Introduction

Chronic gastritis (CG), which is featured by complicated pathophysiological processes, refers to chronic gastric mucosal injury caused by the imbalance between aggressive and defensive factors. The common pathogenic elements include *Helicobacter pylori* (Hp), alcohol, drugs, duodenogastric reflux, duodenal fluid, autoimmunity, etc. [1]. Currently, the occurrence of CG is considered to be correlated with decreased pepsin and gastrin and enhanced oxidative stress, but the exact pathogenesis remains unclear. Clinically, few specific therapeutic agents are available for CG, while monotherapy or combination therapy like acid suppression, promotion of gastric motility, protection of gastric mucosa, and antibiotics are accepted as general CG therapies. In most cases, these treatments can effectively relieve the symptoms of CG for a relatively long period, but they generally have few therapeutic effects on gastric mucosal atrophy and several of them could induce gastric dysfunction, injury, and even cancer [2]. In addition, disease recurrence after drug withdrawal, antibiotic resistance after long-term medication, and several other side effects complicate the treatment of CG [3–5]. Long-term nonatrophic CG is very likely to progress into atrophic CG, which is a recognized precursor of gastric cancer [6].

Ultraviolet C (UVC) irradiation is a common physiotherapy with recognized clinical effectiveness. UVC can significantly reduce superficial aseptic inflammation, mucosal inflammation, and ulcers and promote tissue healing [7–11].

With the invention and application of endoscopic technology, plentiful physical factors, such as microwave, laser, and ultrasound, have been applied to treat tissues and organs inside the human body. This technology also promotes the application of UVC in treating digestive diseases like CG by irradiating UVC into the gastric cavity through the esophagus with the assistance of endoscope.

In this study, we postulated that UVC irradiation could ameliorate CG and validated our hypothesis by establishing a CG animal model. The possible mechanism was also discussed.

We verified our conjecture by detecting interleukin-6 (IL-6), interleukin-1beta (IL-1 *β*), tumor necrosis factor-alpha (TNF-*α*), pepsinogens (PGI, PGII, PGR), gastrin-17 (G-17), reduced glutathione (GSH), catalase (CAT), malondialdehyde (MDA), and superoxide dismutase (SOD), and discussed its mechanism.

## 2. Materials and Methods

### 2.1. UVC Dose

KDY-1 UVC apparatus was produced by the First Medical Center of PLA General Hospital and Beijing Keda Light Source Electric Appliances Co., Ltd. The wavelength ranged from 230 to 280 nm with a 90% percentage of 253.7 nm. The surface power was 1 mW. The quartz derivation was 12 cm long and 3 mm in diameter with a semicircular head.

#### 2.1.1. Detection of UVC Output Dosage

The detection of UVC output dosage was divided into five steps. Firstly, the UV meter was turned on, the wavelength response was set as 254 nm, and the range factor was set as 100. Secondly, the photosensitive surface of the probe was placed in the position to be tested. Thirdly, the UVC apparatus was turned on, the optical output mode was selected, and preheating occurred for 30 sec to stabilize the output dose. Fourthly, the optical head was attached to the light-sensitive probe, UVC exported, the test run for 10 sec, and the test value recorded. Fifthly, the power density was calculated according to the formula: power density (mW/cm^2^) = test value (*μ*W/cm^2^) × range factor (100)/1000.

#### 2.1.2. Test Results

Results were obtained from triplicate experiments. The diameter of the optical head was 3 mm. The area of the optical head was 0.07 cm^2^. According to the formula used in the calculation of clinical biological doses, the output dose of an optical head with an area of 0.34 cm^2^ reaching over 60 mW/cm^2^ is defined as the 1 sec minimal erythema dose (MED). It could be inferred that the output dose of the optical head with an area of 0.07 cm^2^ was 4.1 mW/cm^2^, which was equal to 1/3 MED.

#### 2.1.3. Determination of UVC Dose for the Treatment of CG

The common dose that is used for the clinical treatment of oral mucosal injury or ulcer is 8 MED [12, 13]. Moreover, direct exposure of the optical conductor to the damage surface is required, and radiancy will decrease by 50% when the exposure distance reaches 1 cm away from the damage surface. In this study, UVC irradiation was performed blindly within rat gastric tissues with smaller exposure area than the straight optical conductor. The presence of gastric juice also prevented the irradiation from reaching the mucosal surface precisely. Gastric mucosal injury can occur in both gastric antrum and body. Accordingly, irradiation for 2 yields was selected in this study, including 1-yield irradiation at the gastric fundus and 1-yield irradiation at 1 cm from the gastric fundus. Thus, based on the previous animal experiments and the preliminary results of our team, we finally chose 24 s × 2 yields and 48 s × 2 yields for the treatment of CG in this study [14, 15].

### 2.2. Establishment of Chronic Gastritis Model in Rats

#### 2.2.1. Animals

Fifty healthy SPF Wistar rats (male : female = 1 : 1, 8 weeks old), weighing 200 ± 20 g, were purchased from Medical Animal Laboratory Center, The First Medical Center of PLA General Hospital (certificate number: SCXK (Beijing) 2019-0009). All rats were housed in cages and placed in a clean room with temperatures of 20–23°C, humidity of 50%–60%, and a 12 h dark/light cycle. During the experiment, rats had free access to chow diet and water. The study was conducted under the guidance of the National Regulations Affairs Concerning Experimental Animals and approved by the Ethics Committee of the First Medical Center of PLA General Hospital (No. 2018pkyn049).

#### 2.2.2. Reagents and Chemicals

All chemicals used to induce CG and treatment were obtained from Aoboxing Bio-Technology Co., Ltd. (Beijing, China). The rat immunosorbent rat assay (ELISA) kits of cytokines (IL-6, IL-1*β*, and TNF-*α*), pepsinogens (PGI, PGII), gastrin-17 (G-17), reduced glutathione (GSH), catalase (CAT), malondialdehyde (MDA), and superoxide dismutase (SOD) were all supplied by Jianglai Bio-Technology Co., Ltd. (Shanghai, China). All the chemicals used in the experiment were of reagent grade unless otherwise stated.

#### 2.2.3. Experimental Grouping and Establishment of CG Model in Rats

After the two-week adaptive feeding, fifty rats were randomly divided into five groups (10 rats per group), including normal group (Group A), model group (Group B), 20 mg/kg omeprazole (OMEP) (Group C), 24 s intragastric UVC irradiation group (Group D), and 48 s intragastric UVC irradiation group (Group E). The rats in Group A had free access to chow diet and water. Each rat in Groups B–E was intragastrically administrated with 1 mL anhydrous ethanol weekly for eight weeks and had free access to sodium deoxycholate solution (2%) in place of water. In the next fourteen weeks, these rats were given sodium deoxycholate solution (2%), ethanol (20%), or ammonium hydroxide (0.1%) alternately every two weeks [16, 17]. The rats in Group A were administered with water instead of anhydrous ethanol by gavage.

A stainless steel needle with a diameter of 45 mm was used for gavage. Water bottles were routinely cleaned, and the liquids in the bottles were changed daily. All the rats were fasted and dehydrated for 12 h before and after each gavage or irradiation.

### 2.3. Interventions

All rats had free access to chow diet and water after the successful establishment of the CG model. All the experiments were performed in the morning. All the rats were fasted and dehydrated for 12 h before each experiment.

  Group A: intragastric intubation using the quartz derivation without irradiation for seven constructive days
  Group B: no interventions
  Group C: OMEP (20 mg/kg) for seven constructive days [18]
  Group D: intragastric UVC irradiation for 24 s × 2 yields on day 1 and intubation without irradiation on day 2 to day 7
  Group E: intragastric UVC irradiation for 48 s × 2 yields on day 1 and intubation without irradiation on day 2 to day 7

All rats had free access to chow diet and water after the whole experiment and fasted and dehydrated for 12 h at one week later. Then, these rats were anesthetized by injection with 3% pentobarbital sodium solution (45 mg/kg). Incisions were made downward 4–5 cm from the xiphoid and abdominal aorta was exposed. Blood was collected by puncture from distal to the proximal abdominal aorta and stored in blood-collecting tubes. Sera were collected from whole blood after centrifugation at 3000 rpm for 15 min and stored at −80°C for further analyses. Rats were then sacrificed and gastric tissues were collected and preserved in −80°C for examination of GSH, CAT, SOD, and MDA.

### 2.4. Gastric Histopathological Examination

Rat gastric tissues were sheared along the greater gastric curvature and gently washed with flowing normal saline. The tissue specimens were fixed in 10% formaldehyde solution for 48 h and then dehydrated with gradient alcohol. Then, the specimens were cleared with xylene and immersed and embedded with paraffin.

Four micron thick gastric tissue slices were prepared and stained with hematoxylin and eosin (H&E) using standard methods. The thickness of the gastric wall, the color and luster of gastric mucosa, the number and elasticity of mucosal folds, edema, and gastrorrhagia were observed and recorded. The activity of gastric mucosa, chronic inflammatory response, and gastric mucosal atrophy were classified into four grades based on the degrees of neutrophil infiltration, including normal, mild, moderate, and severe [19]. The four grades were scored as 0 (0 = normal), 1 (1 = mild), 2 (2 = moderate), and 3 (3 = severe), respectively; then, the scores of gastric mucosal activity (scored by neutrophil infiltration in the setting of a chronic inflammatory response), chronic inflammatory response (graded according to the density and depth of the infiltration of chronic inflammatory response cells into the mucosal layer, and under ambiguous conditions, the classification should be based on the cell density), and gastric mucosal atrophy (diagnosed by a 1/3 reduction in the gastric intrinsic glands) were calculated and defined as the indicators for evaluating the efficacies of treatments [20, 21]. The pathological evaluation was performed by two pathologists with more than 10-year experience.

### 2.5. Determination of Serum Interleukin-6 (IL-6), Interleukin-1 Beta (IL-1*β*), and Tumor Necrosis Factor-Alpha (TNF-*α*) Levels

The levels of IL-6 (JL20896), IL-1*β* (JL20884), and TNF-*α* (JL13202) in serum were determined by rat ELISA kits, and the procedures of IL-6, IL-1*β*, and TNF-*α* kits were described in [22].

### 2.6. Measurement of Serum Pepsinogens (PGI, PGII), PGR (PGI/PGII), and Gastrin-17 (G-17) levels

The levels of serum PGI (JL21317), PGII (JL19874), and G-17 (JL45791) were detected using rat ELISA kits [23]. PGR is the ratio of PGI and PGII. According to the instructions, the OD value at 450 nm of the supernatant was quantitatively determined using the PGI, PGII, and G-17 kits.

### 2.7. Detection of GSH, CAT, SOD, and MDA Levels in Rat Gastric Tissues

The activities of SOD (JL22893) and CAT (JL21028) as well as the contents of GSH (L21015), MDA (JL13297), and proteins were measured for evaluating the degrees of lipid peroxidation in rat gastric tissues and detected using rat ELISA kits [24]. Briefly, rat gastric tissues were washed using normal saline and then dried out with a towel. The loading buffer was added based on sample weight. Then tissues were homogenated using a Teflon Homogenizer and centrifuged at 4°C, 10,000 rpm for 60 min, which was stored in a refrigerator at −80°C before experiments. The supernatant was separated for measuring GSH, CAT, SOD, and MDA according to the manufacturer in the kits. The OD values were detected at 450 nm using a spectrophotometer.

### 2.8. Statistical Analysis

For the quantitative data, data are expressed as median, quartile range (M, QR). The differences between groups were compared by one-way ANOVA using SPSS software ver.19 (IBM-SPSS Inc., Chicago, IL, USA). Data are expressed as mean ± standard deviation. Values of *P* < 0.05 were considered statistically significant.

## 3. Results

### 3.1. Establishment of the CG Model in Rats

Three rats died during the establishment of the model (Table 1). The rat in Group B died possibly from the improper experimental operation. The rat struggled hard when grasped by the researcher. Then, its body below its head and neck escaped from the researcher's hands, which caused its death from cervical dislocation. The esophagus of the rat in Group C was punctured by the rough lavage needle and fluid flowed into its trachea. Then, the rat died because of asphyxia. The rat in Group E developed body emaciation and hypotrichosis when it died. Gastric dissection showed severe hyperemia and erosions in the gastric mucosa and disappeared plica mucosa. This could be a result of the model establishment.

### 3.2. UVC Irradiation Relieves Gastric Mucosal Lesions of CG Rats

Most CG rats were characterized by the uneven inelastic gastric wall, pale or dark mucosa, with a small amount of mucus adhered, and disorganized, scarce, or flat gastric folds. Some of the rats developed gastric mucosal hemorrhage and/or edema. Results showed that gastric mucosal injury was significantly relieved in rats which had received UVC irradiation for 48 s × 2 yields. UVC irradiation for 48 s × 2 yields was comparable to OMEP in improving gastric mucosal injury. Macroscopic and histopathological examinations revealed that both UVC irradiations for 24 s × 2 yields and 48 s × 2 yields effectively mitigated ethanol-induced gastric mucosal injury in CG rats, while UVC irradiation for 48 s × 2 yields showed a greater efficacy.

**Figure 1:**
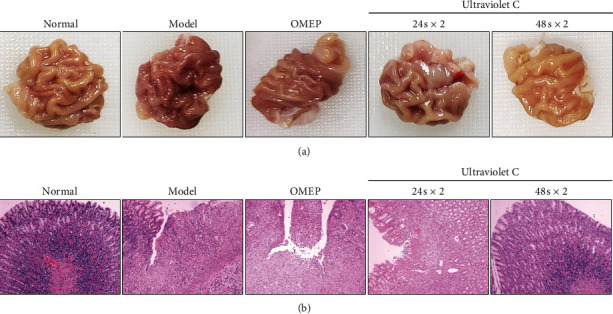
Effect of UVC irradiation on gastric tissues of rats with CG. (a) Gastric morphology. (b) HE staining of the gastric mucosa (×100).

**Figure 2:**
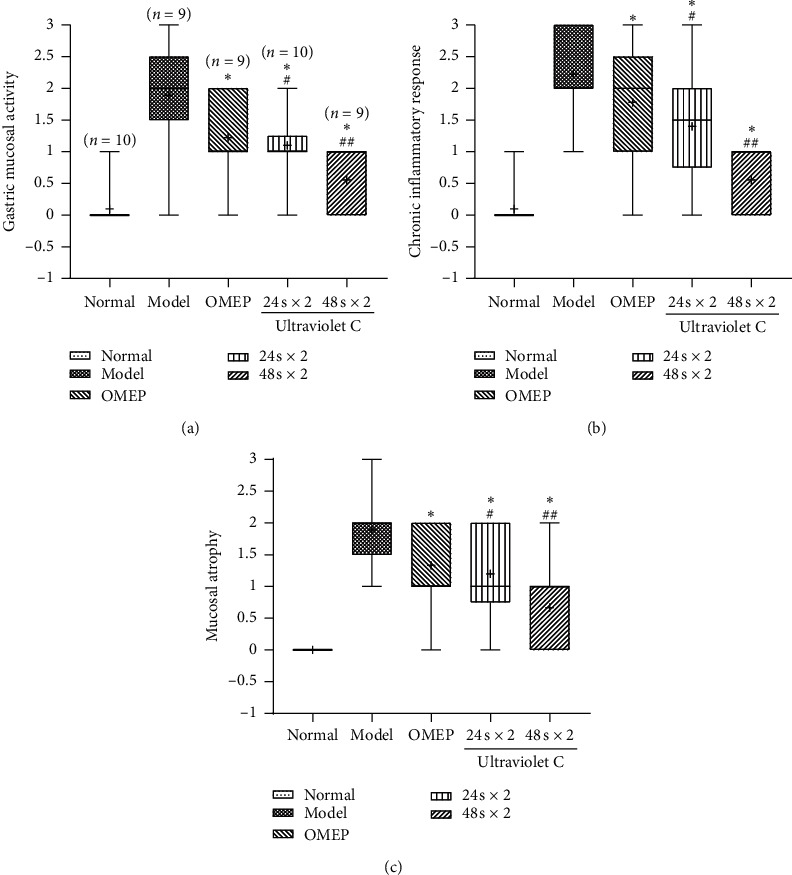
(a) Gastric mucosal activity. (b) Chronic inflammatory response. (c) Gastric mucosal atrophy.  ^*∗*^*P* < 0.05 versus model.  ^#^*P* < 0.05,  ^##^*P* < 0.01 versus OMEP, and  ^##^*P* < 0.01 versus 24 s × 2.

It is of great importance for the evaluation and quantification of inflammation and atrophy during CG to determine the degrees of gastric mucosal activity, chronic inflammatory response, and gastric mucosal atrophy. As shown in Figures 1 and 2 and Table 2, both OMEP administration and UVC irradiation could significantly decrease the degrees of gastric mucosal activity (Figure 2(a), Table 2), chronic inflammatory response (Figure 2(b), Table 2), and gastric mucosal atrophy (Figure 2(c), Table 2). The effects of UVC irradiation for 24 s × 2 yields were comparable to those of OMEP, while the effects of UVC irradiation for 48 s × 2 yields were the strongest.

### 3.3. UVC Irradiation Decreases the Levels of Serum IL-6, IL-1*β*, and TNF-*α* in CG Rats

**Figure 3:**
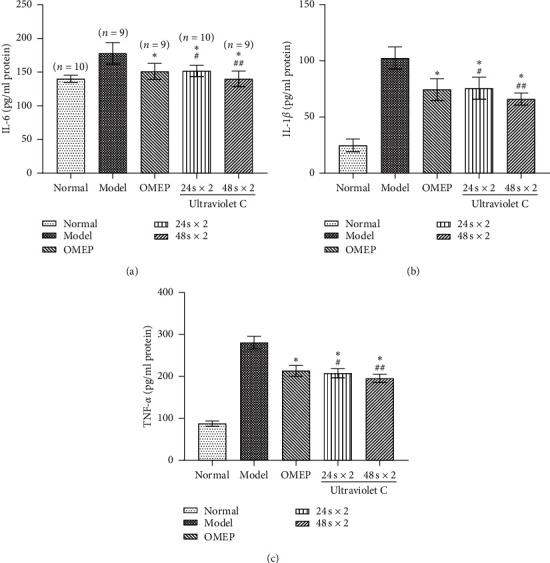
(a) IL-6. (b) IL-1*β*. (c) TNF-*α*.  ^*∗*^*P* < 0.05 versus model.  ^#^*P* < 0.05,  ^##^*P* < 0.05 versus OMEP, and  ^##^*P* < 0.05 versus 24 s × 2.

The serum levels of IL-6 (Figure 3(a)), IL-1*β* (Figure 3(b)), and TNF-*α* (Figure 3(c)) were determined. The levels of TNF-*α* and IL-*β* in model group were higher than those in normal group. 24 s × 2 field irradiation and OMEP had the same effect in reducing the levels of IL-6, IL-1*β*, and TNF-*α*, while 48 s × 2 field irradiation had the most significant effect.

### 3.4. UVC Irradiation Decreases the Levels of Serum PGI, PGII, PGR, and G-17 in CG Rats

**Figure 4:**
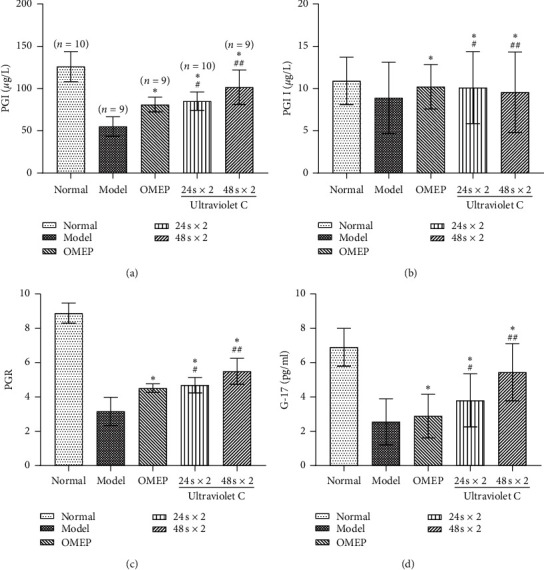
(a) PGI. (b) PGII. (c) PGR. (d) G-17. (a, b, d)  ^*∗*^*P* < 0.01 versus model,  ^##^*P* < 0.01versus 24 s × 2, and  ^#^*P* > 0.05 versus OMEP. (c)  ^*∗*^*P* > 0.05 versus model,  ^#^*P* > 0.05 versus OMEP, and  ^##^*P* > 0.05 versus 24 s × 2.

The levels of serum PGI, PGII, PGR, and G-17 in all rats were measured (Figure 4). Results showed that UVC irradiation had no effects on the level of serum PGII but increased the levels of serum PGI, PGR, and G-17 in CG rats. This could be explained by the different sources and distribution of PGII [25]. The increase in serum PGI levels caused an increase in serum PGR levels. UVC irradiation for 24 s × 2 yields showed equivalent effects to OMEP on increasing the levels of serum PGI, PGR, and G-17 in CG rats. UVC irradiation for 48 s × 2 yields exhibited the strongest ameliorative effect.

### 3.5. Effects of UVC on GSH, CAT, SOD, and MDA in Gastric Tissues of Rats with CG

**Figure 5:**
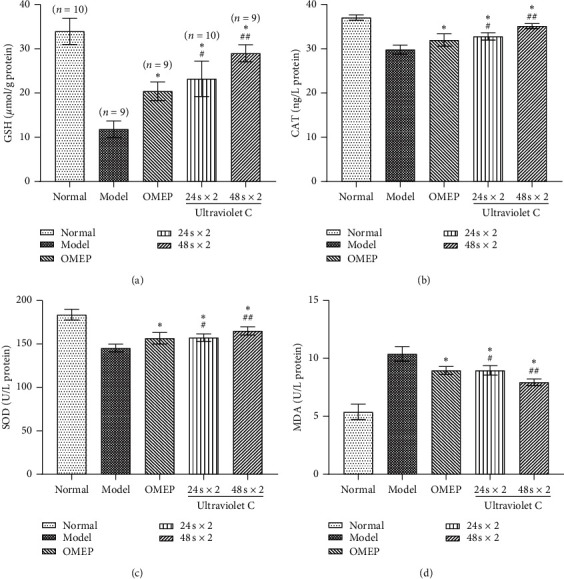
(a) GSH. (b) CAT. (c) SOD. (d) MDA  ^*∗*^*P* < 0.05 versus model.  ^#^*P* > 0.05 versus OMEP.  ^##^*P* < 0.01 versus OMEP, 24 s × 2.

Generally, oxidant and antioxidant systems in organisms are kept under a dynamic balance. However, the development of CG is usually accompanied by enhanced oxidative stress and damaged antioxidant system in gastric tissue [26, 27]. MDA is a biomarker of oxidative stress. GSH, CAT, and SOD eliminated intracellular oxygen free radicals in cells, thereby protecting cells from injury [28]. Herein, we measured the levels of GSH, CAT, SOD, and MDA in rat gastric tissues (Figure 5). Results showed that OMEP significantly increased the activity of CAT and SOD as well as GSH levels and decreased the content of MDA possibly by reducing the secretion of gastric acid and promoting the repair of the gastric mucosa UVC irradiation for 24 s × 2 yields had comparable effects to OMEP on increasing CAT and SOD activity, improving GSH levels, and decreasing MDA content, while UVC irradiation for 48 s × 2 yields showed a stronger antioxidative effect.

## 4. Discussion

The mechanisms of UVC promoting tissue regeneration remained unclear. The existing literature showed that, in addition to the direct disinfection and sterilization effects on local lesions, appropriate UVC irradiation could accelerate and promote tissue repair, including skin, mucosa, and other soft tissues. For instance, short-time low-dose UVC irradiation could induce the photochemical reaction on the skin, enhance cellular metabolism, improve tissue vasospasm, accelerate blood flow, and increase blood perfusion [7]. UVC could also facilitate the synthesis of DNA and RNA and promote mitosis, cell growth, and reproduction. In addition, UVC could stimulate cells to produce and secrete various cytokines, such as IL-6, IL-1*β*, and TNF-*α*, etc. Through the chemotaxis of the cytokines above, granulocytes, mononuclear macrophages, and some other cells could exert their anti-inflammatory effects. Moreover, UVC could suppress inflammatory response and reduce inflammation (some CG cases are induced because of autoimmunity disorder) [29–31].

Although the pro-regenerative effects of UVC have been widely reported, almost no researches focused on its effects on chronic gastric mucosal injury. Herein, this study was aimed at investigating the protective effects of UVC on gastric mucosa and the mechanisms.

Previous reports have confirmed that the combination of ethanol, sodium deoxycholate, and ammonium hydroxide could induce the occurrence of CG [16]. Mucosal atrophy and neutrophil infiltration were typical histopathological alternations of CG, which could be used to determine whether the model was established. OMEP is a commonly-used drug for the treatment of CG, which then was selected as the positive drug in this work [32].

IL-6, IL-1*β*, and TNF-*α*, as important proinflammatory mediators, are often used to evaluate the severity of gastric mucosal injury. Previous studies have found that the levels of TNF-*α* and IL-1*β* in patients with chronic gastritis are higher than those in normal people, and their levels increase with the aggravation of gastric mucosal lesions, suggesting that these inflammatory factors may play a role in aggravating the inflammatory response to chronic gastritis. In our research, UVC can significantly inhibit the serum IL-6, IL-1*β*, and TNF-*α* of the model rats, and reduce the inflammatory reaction.

According to the consensus on chronic gastritis in China published in 2017 [19], the levels of serum PGI, PGII, PGR, and G-17 could be used to accurately distinguish the secretion features, atrophy degrees, and tissue function of gastric mucosa in different areas. Thus, the measurement of these markers has been recognized as a reliable noninvasive method for diagnosing gastric mucosal diseases in early stages [6, 33]. Several previous studies indicated that the levels of PGI and PGR were positively correlated with the severity of corpus gastritis [34–37], while the level of G-17 decreased with the aggravation of antral inflammation and atrophy [38–40]. This study demonstrated that UVC irradiation could not only increase the levels of serum PGI and PGR but also increase the G-17 levels. These results suggested that irradiation with UVC of different doses for 2 yields could fully cover gastric corpus and antrum, which further alleviated chronic gastric inflammation and promoted the repair of atrophic mucosa.

In this study, our results suggested that UVC irradiation could increase CAT and SOD activity as well as GSH levels and decrease MDA content to enhance the antioxidative ability of rats with CG induced by physiochemical irritants. These results indicated that UVC irradiation could restore the balance between aggressive and defensive factors by modulating the antioxidative function, which further protected gastric mucosal cells from the attack by free radicals, thereby relieving gastric tissue injury.

## 5. Conclusions

This study showed that UVC irradiation, especially 48 s × 2 yields, could effectively improve gastric mucosal activity, inflammatory response, and atrophy, inhibit inflammation factor, increase the contents of PGI, PGR, and G-17, improve the antioxidative effect of gastric mucosa, and protect gastric mucosa of rats with CG induced by physiochemical irritants. Our current results suggested that UVC could be considered as a promising novel therapeutic strategy for CG.

## Figures and Tables

**Table 1:** The mortality of CG rats.

Groups	*n*	Mortality
Normal	10	0
Model	10	1
OMEP	10	1
Ultraviolet C (24 s × 2)	10	0
Ultraviolet C (48 s × 2)	10	1

One CG model rat, one OMEP-treated rat, and one rat receiving ultraviolet C irradiation for 48 × 2 yields died during the experiment.

**Table 2:** The degrees of gastric mucosal activity, chronic inflammatory response, and gastric mucosal atrophy (M, QR).

Groups	*n*	Gastric mucosal activity	Chronic inflammatory response	Gastric mucosal atrophy
Normal	10	0.00, 0.00	0.00, 0.00	0.00, 0.00
Model	9	2.00, 1.00	2.00, 1.00	2.00, 1.00
OMEP	9	1.00, 1.00 ^*∗*^	2.00, 2.00 ^*∗*^	1.00, 1.00 ^*∗*^
Ultraviolet C (24 s × 2)	10	1.00, 0.00 ^*∗#*^	1.50, 1.00 ^*∗#*^	1.00, 1.00 ^*∗#*^
Ultraviolet C (48 s × 2)	9	1.00, 1.00 ^*∗##*^	1.00, 1.00 ^*∗##*^	1.00, 1.00 ^*∗##*^

Data are expressed as median, quartile range (M, QR).  ^*∗*^*P* < 0.05 versus model,  ^#^*P* < 0.05 versus OMEP, and  ^##^*P* < 0.05 versus 24 s × 2.

## Data Availability

The data used to support the findings of this study are available from the corresponding author upon request.
